# Clinical and epidemiological criteria for idiopathic granulomatous mastitis among women in Sulaymaniyah, Iraq: A retrospective study

**DOI:** 10.1097/MD.0000000000049343

**Published:** 2026-06-19

**Authors:** Mezjda Ismail Rashaan, Bawan Sabir Hamid, Mohammed Jamal Rashid, Rabar Abubakr Hawrami

**Affiliations:** a Branch of Clinical Sciences, College of Medicine, University of Sulaimani, Sulaimaniyah, Iraq.

**Keywords:** breast inflammation, breastfeeding, clinical correlation, clinical presentation, granulomatous mastitis

## Abstract

Idiopathic granulomatous mastitis (IGM) is a chronic, rare, non-cancerous, non-lactational mastitis that mostly occurs in parous women with a history of breastfeeding. To identify the sociodemographic, clinical presentation, etiological factors, and the possible clinical correlations among female patients with IGM, using clinical and epidemiological criteria. This observational, retrospective, cross-sectional study evaluates 100 patients with IGM, from 2015 to 2025 at the Breast Center, Shar Teaching Hospital, Sulaimaniyah, Iraq. Their sociodemographic characteristics, reproductive history, clinical presentation, and clinical correlations to the development of IGM were collected and analyzed using a standard questionnaire. The mean age of the patients was 36.5 ± 7.8 years. The majority has normal body weight (46%), residence in urban area (91%), illiterate (45%), married (99%), medium class (69%), housewives (73%), smokers (67%), had 3-5 children (44%), had no physical activity (79%), had a history of last breastfeeding < 5 years (63%), had breastfed > 12 months (53%), had bra-wearing habits (93%), practiced hygienic breastfeeding (97%), and had a unilaterally affected breast (99%), especially the upper outer quadrant (UOQ; 38%). The profound clinical presentations were breast mass (96%), pain (86%), and erythema (52%). Also, 45% had thyroid disease, 11% had diabetes, 33% used hormones, 31% had abnormal sleep, and 29% had an emotional abnormality. IGM is a chronic inflammatory disease of obscure etiology that occurs mainly in parous women with a history of prolonged breastfeeding. Its clinical characteristics are a unilateral breast mass with or without pain, mostly at the UOQ. Exogenous hormonal users, psychotropic drugs, hyperprolactinemia, and thyroid disorders are shown to have a role in developing IGM.

## 1. Introduction

Idiopathic granulomatous mastitis (IGM) is a non-puerperal mastitis, an uncommon chronic inflammatory granulomatous breast disease, of unknown etiological factors and easy recurrence.^[1]^ Generally, IGM affects young women of reproductive age and rarely occurs in males.^[2]^ Because of the characteristic histology of the lesion, it is currently recommended to replace IGM with granulomatous lobular mastitis.^[3]^ It has an annual prevalence of 2.4 in 100,000 and a 0.37% incidence rate in the population.^[4]^ The IGM incidence rate is about 1.8% in all mammary diseases and 24% in inflammatory breast disease. The prevalence of IGM is higher in Middle Eastern, Mediterranean region, Asian, and Hispanic populations than in Western Caucasian races.^[5]^ IGM is a disease of a long course with relapses; it poses a considerable effect on the patient’s breast appearance causing disfigurement, quality of life, mental health and health burden on childbearing women.^[6]^

Most affected IGM patients are parous women around 35 years old and may have breastfed within 5 years of diagnosis.^[4]^ Although IGM etiology is unclear, some conditions have been postulated as predisposing factors, including reproductive age, pregnancy, lactation, oral contraceptive pills (OCP), hyperprolactinemia, infections, trauma, and autoimmune disease.^[7]^ Currently, different theories are contributing to the occurrence of IGM. The most accepted theory considers IGM to be a humoral and cellular-mediated immune reaction that is stimulated by extravasated intraductal protein-rich secretion and milk through increasing mammary duct permeability to the mesenchymal tissue of the mammary lobules and causing nonspecific lobulitis. The increasing permeability of the mammary ducts may be stimulated by hormonal imbalance, trauma, duct ectasia, microbiological agents, and retained milk.^[8]^

Usually, IGM occurs unilaterally with no tendency to a side, rarely bilaterally, may affect all quadrants of the breast, and may be uni- or multifocal.^[9]^ The early clinical manifestation of IGM is an ill-defined breast mass with or without pain, local skin changes, and signs of a systemic disease unless complicated.^[10]^ But in the advanced stage, skin ulceration, purulent discharge, abscess, sinus, and fistula formation occur.^[11]^ Axillary lymphadenopathy occurs in up to 15% of patients^[2]^ that mimics breast cancer (BC).^[7]^ However, no association and risk have been reported between IGM and BC.^[4]^ Concomitant systemic involvement may occur at any stage, such as arthralgia, erythema nodosum, oligoarthritis, and episcleritis, which may favor the IGM diagnosis.^[9]^ The cornerstone to confirm the definitive diagnosis of IGM is by histopathological examination, as IGM simulates BC clinically and radiologically.^[5]^

It is recommended in suspected cases of IGM to check routine blood tests, C-reactive protein, antinuclear antibody profile, rheumatism factor, serum prolactin level, and purified protein derivative of tuberculin test.^[1]^ Currently, there is no worldwide unified guideline and no ideal treatment for IGM patients.^[12]^ Thus, we aimed to find the sociodemographic, clinical presentation, etiological factors, and the possible clinical factors associated with IGM development, using clinical and epidemiological criteria.

## 2. Patients and methods

### 2.1. Study design and setting

In this observational, retrospective, cross-sectional study, the data of 100 female patients with IGM from January 2015 to January 2025 were collected from the Breast Center, Shar Teaching Hospital, Sulaimaniyah, Iraq, using a consecutive sampling method. Baseline data were obtained from patients’ file records in the hospital together with a direct patient contact via phone calls.

### 2.2. Inclusion criteria

Female patients with IGM, who were initially diagnosed based on signs, symptoms, medical history, and physical examination, then confirmed by imaging (ultrasound of the breast and mammography) and histopathological examination (tru-cut biopsy or wall biopsy).

### 2.3. Exclusion criteria

Patients diagnosed with or suspected to have BC. Also, those with incomplete medical records or who didn’t respond to phone calls.

### 2.4. Study protocol

A validated standard questionnaire was used to collect patients’ sociodemographic characteristics, reproductive and obstetric history, breastfeeding and exogenous hormonal use, emotional disorders, clinical manifestations, and investigations.

### 2.5. Ethical consideration

Ethical approval was obtained from the Scientific and Ethical Committees of the College of Medicine, University of Sulaimani (No. 209 on May 05, 2024), as well as from the Institutional Review Board of the Shar Teaching Hospital, Sulaimaniyah, Iraq. Written informed consent was obtained from each participant before starting the study, while their confidentiality and anonymity were protected throughout the study.

### 2.6. Statistical analysis

The data were analyzed using SPSS (IBM, Chicago, version 26) with descriptive statistics such as frequency and percentage for categorical variables, and mean and standard deviation for numerical variables.

## 3. Results

The mean age of patients was 36.5 ± 7.8 years (ranging from 23–58 years). Most patients were aged 30 to 40 years (48%), illiterate (45%), from an urban area (91%), married (99%), housewives (73%), had a medium economy (69%), had normal weight (46%), and were smokers (67%) (Table 1).

**Table 1:** Sociodemographics of patients with idiopathic granulomatous mastitis.

Variables	Frequency	Percentage
Age (yr)		
<30	22	22
30–40	48	48
>40	30	30
Body mass index (kg/m^2^)		
Normal weight	46	46
Overweight	38	38
Obese	16	16
Residency		
Urban	91	91
Rural	9.0	9.0
Occupation		
Non employed (free work)	16	16
Housewife (not working)	73	73
Employed	7.0	7.0
Others	4.0	4.0
Educational level		
Illiterate	45	45
Primary and secondary school	34	34
University and Institution	21	21
Marital state		
Married	99	99
Unmarried	1.0	1.0
Economic state		
Low	30	30
Medium	69	69
High	1.0	1.0
Miscellaneous		
Menopause	7.0	7.0
Smokers	67	67
Physical activity	21	21
Alcoholism	1.0	1.0

**Figure 1.**
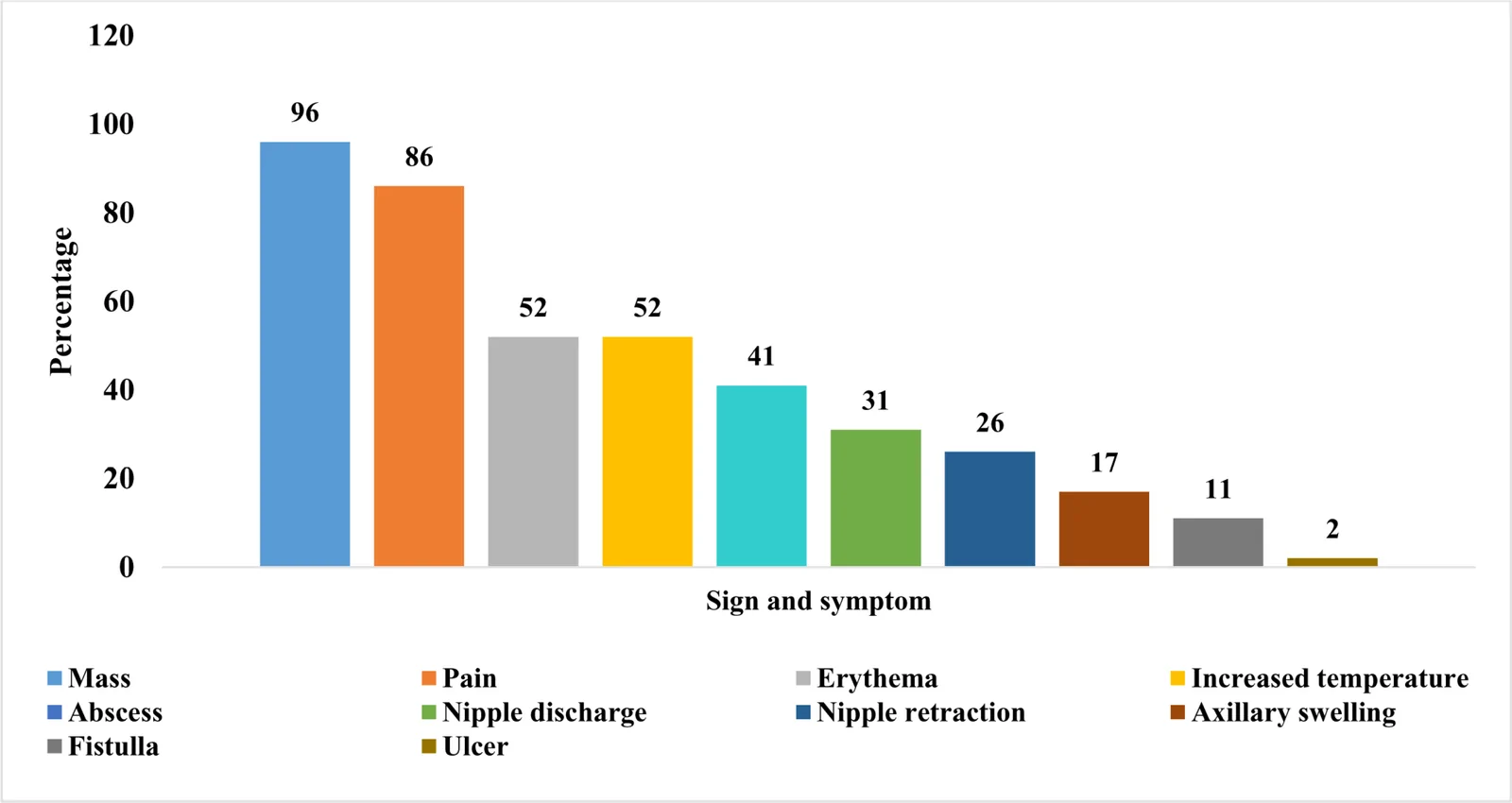
Signs and symptoms of patients with idiopathic granulomatous mastitis.

Majority (44%) had 3 to 5 parities, and the age of their last child at IGM occurrence was between 4 to 5 years (45%). At the time of IGM diagnosis, 64% of the patients were breastfeeding less than 5 years ago, and the breastfeeding duration for most of them (53%) was more than 1 year. Also, most of them practiced hygienic breastfeeding (97%) (Table 2). Interestingly, 18% had a history of breast disease (13% BC and 5.0% IGM), 9.0% had a history of breast lumpectomy for fibroadenoma (6.0% had an operation incision and drainage for breast abscess, and 3.0% had mastitis treated conservatively). Notably, 35% had an abortion history, 20% had a history of breast trauma, 6.0% had a history of autoimmune disease, and 9.0% had drug and/or food allergy (Table 3). About 33% of patients were hormonal users with a mean duration of 33.12 ± 36.14 months, 9.0% had hyperprolactinemia, 40% had hypothyroidism, 5.0% had hyperthyroidism, 11% had diabetes mellitus (DM), 31% had abnormal sleep, 29% had emotional abnormality, and 93% had bra-wearing habits (Table 4). The site of the disease in most patients (52%) was on the left, 47% on the right, 1.0% bilateral, and 16% multifocal. The most affected quadrant was the upper outer quadrant (UOQ) of both breasts (n = 38), followed by the subareolar area (n = 18) (Table 5). Most patients (n = 96) had a mass, pain (n = 86), erythema/fever (n = 52), followed by abscess (n = 41), nipple discharge (n = 31) (left = 15%, right = 15%, and both sides=1.0%), nipple retraction (n = 26) (new = 15%, old = 11%), axillary swelling (n = 17), fistula (n = 11) (left = 6.0%, right = 4.0%, and both sides = 1.0%), and ulcer (n = 2.0) (Fig. 1).

**Table 2:** Clinical features of patients with idiopathic granulomatous mastitis.

Variables	Number	Percentage
Parity		
0.0	1.0	1.0
1–2	39	39
3–5	44	44
>5	16	16
Age of last child at the time of diagnosis (yr)		
0–2	9.0	9.0
2–4	9.0	9.0
4–5	45	45
>5	36	36
Diagnosis regarding last breastfeeding (yr)		
<5	63	63
>5	36	36
Last breastfeeding duration (mo)		
None	9.0	9.0
0–3	2.0	2.0
3–6	8.0	8.0
6–12	28	28
>12	53	53
Breastfeeding hygiene		
Yes	97	97
No	3	3

**Table 3:** Personnel and family history of patients with idiopathic granulomatous mastitis.

Variables	Frequency	Percentage
Pervious history of breast disease	18	18
Fibroadenoma (lumpectomy)	9.0	9.0
Abscess (incision & drainage)	6.0	6.0
Mastitis (conservative treatment)	3.0	3.0
Family history of breast disease	18	18
Breast cancer	13	13
Idiopathic granulomatous mastitis	5.0	5.0
History of abortion	35	35
Breast trauma history	20	20
History of autoimmune disease	6.0	6.0
History of drug and/or food allergy	9.0	9.0

**Table 4:** Hormonal, emotional, and other states of the patients with idiopathic granulomatous mastitis.

Variables	Frequency	Percentage
Hormonal use	33	33
Hyperprolactinemia	9.0	9.0
Thyroid disease	45	45
Diabetes mellitus	11	11
Abnormal sleep condition	31	31
Emotional abnormality	29	29
Bra wearing habits	93	93
Using local herb	1.0	1.0

**Table 5:** The site and location of breast lesions among patients with idiopathic granulomatous mastitis.

Variables	Number (percentage)
The site	
Left	52 (52)
Right	47 (47)
Bilateral	1.0 (1.0)
Location	
Upper outer quadrant	38 (38)
Upper inner quadrant	19 (19)
Lower outer quadrant	16 (16)
Lower inner quadrant	0.0 (0.0)
Subareolar	18 (18)

## 4. Discussion

Although the incidence rate of IGM is increasing, the predisposing factors and etiology are still not fully understood, which would guide specific clinical management, disease prevention strategies, and improve the quality of life for susceptible women.^[11]^ Moreover, the mechanism of the predisposing factors for IGM, which are linked to various agents and situations such as pregnancy, lactation, contraceptive pill use, hyperprolactinemia, local irritants, infectious agents, smoking, and systemic immunological disorders, are not very clear.^[13]^

In the current study, IGM mainly occurred in those aged 30 to 40 years (48%) with a mean age of 36.5 ± 7.8 years. These outcomes are close to those of another study which found the mean age of the patients to be 33.14 ± 6.71 years.^[12]^ Also, most patients with IGM were from urban areas (91%), housewives (73%), had a medium social class (69%), and had no education certificate (45%). These outcomes are comparable to some other studies.^[10,13,14]^

Most patients (46%) had normal weight, which is not aligned with other studies.^[15–17]^ Obesity causes hormonal changes, especially in estrogen, which may influence breast tissue and cause low-grade inflammation. IGM was most common among smokers (67%). While smoking is among the factors enhancing IGM development, no definitive conclusion between smoking and IGM has yet been established.^[17]^ Among study patients, 21% performed physical activity, 1.0% were alcoholic, and 1.0% used local herbs. The significance of such factors is unknown and needs further studies. Bra-wearing was a habit in 93%, while in the previous study, it was 78.2%.^[18]^ This is an independent factor and needs further evaluation to determine if this minor trauma of friction and a tight bra has any effect on the breast tissue.

In this study, 99% of patients were married, which is analogous to previous studies.^[12,17]^ Childbirth was 99%, and those who had ≥2 children were 87%, which is parallel to another study (82.7%).^[19]^ At the time of IGM diagnosis, 63% of the patients had last breastfed < 5 years ago, and 36% were >5 years ago; similar results were reported in a previous study.^[9]^ The age of the last child at the time of diagnosis was 2 to 5 years in 54%, which is analogous to another study (61.8%).^[18]^ Also, 53% had breastfed for >12 months, which is close to another study (56.3%).^[20]^ Frequently, patients neglect routine breast massage and complete evacuation of both breasts after lactation. During the first weeks of weaning, they depend on lactation from one breast, which may lead to milk stasis and hyperprolactinemia that enhance the development of IGM.^[6]^ It is suggested that multiple pregnancies and breastfeeding episodes increase mammary duct permeability, which is a high-clinical factor for the development and recurrence of IGM.^[5]^ Additionally, 97% of patients in this study practiced hygienic breastfeeding. IGM is frequently considered a noninfectious autoimmune condition; however, it has a well-documented relationship with subsequent infection, especially *Corynebacterium* species.^[18]^ Poor hygienic practices during breastfeeding might introduce pathogens and lead to infectious complications.^[4]^ In this regard, Gondkar et al, 2024 considered poor hygiene as an enhancing factor for bacterial infection and IGM development, and they suggest cleaning the breast properly before and after feeding.^[21]^

This study has similar ethnicity, race, and region, mostly found in Mediterranean and developing countries. This may explain the association between IGM and prolonged exposure to sex hormones due to multiple parity, prolonged breastfeeding habits, and using OCP, genetic factors, and dietary habits.^[10]^ Interestingly, 18% had a family history of breast disease (13% BC, 5.0% IGM). Although the clinical manifestation of IGM mimics BC, no association and risk have been reported between IGM and BC.^[3]^ In this study, the history of positive abortion was 35%, which is comparable to another study (47.77%).^[17]^ But until now, the significance of this factor has not been reported in the literature. Also, 29% had a trauma history of the breast, but in the literature, it is 45.37%, which is not analogous with the current research.^[17]^ It is reported that physical trauma to the breast is an independent triggering factor for IGM, as trauma causes local mammary duct damage, which stimulates a hypersensitive inflammatory response through cellular or humoral immunity to induce IGM.^[5]^ Consequently, 6.0% and 9.0% had a history of autoimmune disease and drug and/or food allergy, respectively. Another study reported that the frequency of autoimmune disease, erythema nodosum, and arthritis was 7.8 to 20.9%.^[5]^ This autoimmune disease association with IGM patients, good response to steroids, immunosuppressive therapy, and those who had recurrence after surgery, is suggestive that IGM has an immunological basis.^[2]^

The mean age of menarche in this study is 13.1 years, which might be due to the hormonal changes during menstruation triggering or activating IGM in susceptible individuals. The regularity of the menstrual cycle in the present study is 74%, and it is similar to another study (73.4%).^[14]^ Also, 43% of patients were hormonal users. The association of OCPs with IGM as an enhancing clinical factor is 5 to 42%.^[20]^ However, it is not clear how exogenous sex hormones act as a potential clinical factor for developing IGM. While it is suggested that exogenous sex hormones could affect mammary duct secretion and its expansion.^[4,20]^ Additionally, 9.0% had hyperprolactinemia, which is lower than that reported in another study (34.3%).^[5]^

Furthermore, 45% of our patients had thyroid disease, which is lower than that reported in other studies.^[2,16]^ The association between granulomatous thyroiditis and IGM had been reported in a previous study.^[12]^ It is suggested that breast cells are responsive to thyroid hormone changes.^[16]^ However, to understand the relevant mechanism, further studies are necessary. DM rate was low among IGM patients, similar to that in other studies.^[2,12,17]^ This low rate of DM might be due to the participants’ young ages.^[17]^ Moreover, 31% of our patients using psychotropic drugs for abnormal sleep and emotional abnormality. This finding was analogous with the reported studies, which show that patients using psychotropic drugs are at a higher possibility to develop IGM.^[12,17]^ It was reported that the side effects of such drugs cause hyperprolactinemia, increase mammary duct secretion, and trigger an inflammatory autoimmune response through stimulating proinflammatory cytokines (TNF-α and IL-6).^[22]^

Tayler et al detected *Corynebacterium* infection in breast lesions of IGM, in which 14.1% were *Corynebacterium* kroppenstedtii, the normal endogenous flora of breast skin. It is reported that bacteria go deeper into breast tissue through mammary ducts.^[10]^ The clinical manifestations of IGM patients were more common in the left breast, mostly at the UOQ of the breast, but rarely bilateral. These findings are analogous with some reported studies.^[12,18]^ The most common clinical presentations of IGM were unilateral painful breast mass and erythema. These clinical findings verified the reports of previous studies.^[1,2,23]^ In this study, 41% developed abscess and 11% fistula, which is similar to other studies.^[4,5]^ Patients had nipple discharge (31%), which is higher than that of another study (15.6%), which was an independent clinical factor for IGM.^[17]^ The patients’ nipple retraction was 26% that was higher than that of another study (17.7%).^[17]^ However, it is suggested that nipple retraction causes narrowing and obstruction of the mammary ducts, leading to an increase in intraductal pressure and causing ductal damage, which stimulates an autoimmune response that promotes IGM. Axillary lymphadenopathy was predominantly negative, which was similar to other studies.^[11]^

## 5. Conclusions

In this retrospective cross-sectional study, IGM was most common among childbearing ages, those with multiple parity, and those who had prolonged breastfeeding. The most common clinical manifestation was a unilateral painful mass, frequently at the UOQ and areola. Local skin changes occurred later in the course of the disease. Nipple retraction was clinically correlated to IGM. Using exogenous hormonal therapy for menstrual cycle abnormalities, OCP, and psychotropic drugs for negative emotional and sleep might be associated with a high incidence of IGM development. Therefore, these clinically associating factors might be preventable by more supportive and preventive measures, promoting a good mood and using fewer pharmacological drugs. The limitations of this study include its retrospective analysis of a single center-based, and small sample size. Also, there was bias in patient’s information file due to limited data collection and low educational state of included patients as they were not informative about the disease. Finally, financial limitations as hormonal level, blood tests for autoimmune disease markers and culture for infection not performed. Consequently, future studies should include prospective longitudinal design, multi-centered, and enrolling larger sample size to corroborate these results and improve their relevance.

## Acknowledgments

The authors thank the healthcare staff of the Breast Center at Shar Teaching Hospital, Sulaimaniyah, Iraq, as they facilitated the data collection and the cooperation of the included patients. Also, the authors have no conflict of interests and not received any funding supports, locally or internationally.

## Author contributions

**Conceptualization:** Mezjda Ismail Rashaan.

**Data **acquisition**:** Rabar Abubakr Hawrami.

**Formal analysis:** Bawan Sabir Hamid.

**Methodology:** Bawan Sabir Hamid.

**Project administration:** Mohammed Jamal Rashid.

**Visualization**: Mohammed Jamal Rashid.

**Supervision:** Mezjda Ismail Rashaan.

**Validation:** Mezjda Ismail Rashaan.

**Writing – original draft:** Bawan Sabir Hamid, Mohammed Jamal Rashid, Rabar Abubakr Hawrami.

**Writing – review & editing:** Mezjda Ismail Rashaan.
